# Including Measures of High Gamma Power Can Improve the Decoding of Natural Speech From EEG

**DOI:** 10.3389/fnhum.2020.00130

**Published:** 2020-04-29

**Authors:** Shyanthony R. Synigal, Emily S. Teoh, Edmund C. Lalor

**Affiliations:** ^1^Department of Biomedical Engineering, University of Rochester, Rochester, NY, United States; ^2^Trinity Centre for Biomedical Engineering, School of Engineering, Trinity College Dublin, University of Dublin, Dublin, Ireland; ^3^Trinity College Institute of Neuroscience, Trinity College Dublin, University of Dublin, Dublin, Ireland; ^4^Department of Neuroscience and Del Monte Institute for Neuroscience, University of Rochester, Rochester, NY, United States

**Keywords:** EEG, temporal response function (TRF), high gamma power, decoding attention, speech envelope

## Abstract

The human auditory system is highly skilled at extracting and processing information from speech in both single-speaker and multi-speaker situations. A commonly studied speech feature is the amplitude envelope which can also be used to determine which speaker a listener is attending to in those multi-speaker situations. Non-invasive brain imaging (electro-/magnetoencephalography [EEG/MEG]) has shown that the phase of neural activity below 16 Hz tracks the dynamics of speech, whereas invasive brain imaging (electrocorticography [ECoG]) has shown that such processing is strongly reflected in the power of high frequency neural activity (around 70-150 Hz; known as high gamma). The first aim of this study was to determine if high gamma power scalp recorded EEG carries useful stimulus-related information, despite its reputation for having a poor signal to noise ratio. Specifically, linear regression was used to investigate speech envelope and attention decoding in low frequency EEG, high gamma power EEG, and in both EEG signals combined. The second aim was to assess whether the information reflected in high gamma power EEG may be complementary to that reflected in well-established low frequency EEG indices of speech processing. Exploratory analyses were also completed to examine how low frequency and high gamma power EEG may be sensitive to different features of the speech envelope. While low frequency speech tracking was evident for almost all subjects as expected, high gamma power also showed robust speech tracking in some subjects. This same pattern was true for attention decoding using a separate group of subjects who participated in a cocktail party attention experiment. For the subjects who showed speech tracking in high gamma power EEG, the spatiotemporal characteristics of that high gamma tracking differed from that of low-frequency EEG. Furthermore, combining the two neural measures led to improved measures of speech tracking for several subjects. Our results indicated that high gamma power EEG can carry useful information regarding speech processing and attentional selection in some subjects. Combining high gamma power and low frequency EEG can improve the mapping between natural speech and the resulting neural responses.

## Introduction

Scalp-recorded electroencephalography (EEG) provides a non-invasive means of investigating cortical activity with high temporal resolution. This makes it particularly suited for studying neural processes such as speech perception—where humans rapidly convert mechanical vibrations of the air into meaning. In terms of speech, the slow varying acoustic envelope of continuous natural speech was found to be reflected in EEG (Luo and Poeppel, 2007; Lalor and Foxe, 2010) which is valuable because speech modulations in the 4–16 Hz range have been shown to contain the most important information regarding intelligibility (Drullman et al., 1994). As a result, many studies tend to focus their analysis around this frequency range when using EEG or magnetoencephalography (MEG) to investigate cortical tracking of the speech envelope (Ahissar et al., 2001; Aiken and Picton, 2008; Peelle and Davis, 2012; Di Liberto et al., 2015).

In contrast to the emphasis on lower-frequency bands in EEG speech research, studies that employ electrocorticography (ECoG) often look at signals in the high gamma range (~70–150 Hz). High gamma ECoG has also been shown to track the speech envelope (Pasley et al., 2012; Kubanek et al., 2013), even though high gamma and low frequency (LF) activity are thought to result from distinct physiological mechanisms (Edwards et al., 2009). The fidelity of speech tracking in high gamma power (HGP) ECoG data is so high that most ECoG studies focus exclusively on that frequency range and ignore the data at lower frequencies. Meanwhile, the high-frequency content of scalp-recorded EEG is typically disregarded because it is low pass filtered by the skull (Pfurtscheller and Cooper, 1975) and smeared by the dura and cerebrospinal fluid (Light et al., 2010), thus resulting in a low signal-to-noise ratio. Nevertheless, we questioned whether there is still useful stimulus-related information dissociable from low-frequency data that could be retrieved from high gamma EEG. If so, high gamma EEG could serve as a useful measure for studying speech and language processing in various populations.

To our knowledge, as this study represented the first effort to do this, we restricted our focus to examining the aforementioned tracking of the temporal speech envelope in HGP EEG. EEG data were recorded as subjects listened to continuous natural speech, and we mapped the EEG (filtered into LF, HGP, and both signals combined) to the temporal speech envelope using linear regression. In a second data set, we investigated whether the inclusion of HGP can improve auditory attention decoding. It is established that envelope tracking in LF EEG is modulated by attention (Kerlin et al., 2010; Power et al., 2012), and cortical HGP has also been shown to be strongly modulated by attention (Mesgarani and Chang, 2012; Zion Golumbic et al., 2013; Dijkstra et al., 2015). Here, we employed a framework that has been successful in ascertaining attentional selection within the context of a task in which subjects attend to one of two concurrent talkers (O’Sullivan et al., 2015). We compared how well attentional selection can be decoded from EEG when using LF, HGP, and a combination of the two. In doing so, we found that for a minority of subjects, the speech envelope and attention are reflected in HGP EEG in a way that may be complementary to the information available in LF EEG.

## Materials and Methods

### Subjects

Two experimental paradigms were explored in the present study using data from three previously published studies. The first paradigm involved subjects listening to a single speaker and the second involved subjects attending to one of two concurrently presented speakers. Data used in the single speaker paradigm originated from two previous studies examining semantic dissimilarity and phoneme level processing (Broderick et al., 2018; Di Liberto et al., 2018)^1^. Seventeen subjects (min = 19 years, max = 31 years, 12 males) were used in total. These studies were approved by the Ethics Committees of the School of Psychology at Trinity College Dublin and the Health Sciences Faculty at Trinity College Dublin. Data used in the attentional selection or cocktail party paradigm was from the control condition of a study which investigated the decoding of auditory attention (Teoh and Lalor, 2019). Fourteen subjects (min = 19 years, max = 30 years, 5 male) took part in the experiment. This study was approved by the Research Subjects Review Board at the University of Rochester. All subjects were native English speakers, provided written informed consent, and reported no history of hearing impairment or neurological disorders.

### Stimuli and Procedure

The single speaker experiment consisted of subjects listening to 20–29 trials (approximately 180 s in length) of a mid-20th century audiobook read by one American male speaker (Hemingway, 1952). The subjects were tested in a dark, sound-attenuated room and were instructed to attend to a fixation cross in the center of a screen. The storyline was preserved in the trials, with no repetitions or discontinuities present. In the multi-speaker experiment, subjects undertook 20 trials (approximately 60 s in length). They were presented with two stories (Doyle, 1892, 1902) simultaneously, narrated by a male and a female speaker. The two audio streams were filtered using head-related transfer functions to simulate spatial separation of the speakers (one speaker at 90 degrees to the left and the other at 90 degrees to the right). The attended speaker was on the left in 50% of the trials and was on the right the other 50%. The subjects were instructed to attend to the male speaker in all trials and to minimize motor movements by fixating to a cross at the center of a screen. Subjects then answered four multiple-choice questions on the attended and unattended stories after each trial (which were not analyzed in this work). In all experiments, the stimuli were presented through Sennheiser HD650 headphones at a 44.1 kHz sampling rate using Presentation software from Neurobehavioral Systems^2^.

### Data Acquisition and Preprocessing

For both experiments, EEG data were acquired at a 512 Hz sampling rate with the BioSemi Active Two system using 128 scalp electrodes (plus two mastoid channels that were not analyzed in this work). Each subject’s scalp data were re-referenced to the common average. Noisy channels were determined based on three of EEGLAB’s artifact rejection methods (kurtosis, spectral estimates, and probability distribution; Delorme and Makeig, 2004), and spline interpolation was used to reject and recalculate the data in those channels. The EEG was then filtered into two separate bands. The first band (low-frequency band) was high pass filtered at 1 Hz and then low pass filtered at 15 Hz using a zero-phase type 2 Chebyshev filter. For the second band, the raw data were bandpass filtered in the high gamma range from 70 to 150 Hz using a 200th order zero-phase FIR filter with a hamming window. The absolute value of the Hilbert transform was taken from the high gamma EEG to extract the power (as is typically done in ECoG studies). The resulting data was then low pass filtered at 15 Hz to match the power spectrum of the LF EEG and to ensure we did not artificially render the LF EEG and HGP EEG differentially sensitive to different features in the speech envelope; this way, decoding using both frequency bands could be more directly compared. Finally, all data were downsampled to 128 Hz.

### Data Analysis

**Figure 1:**
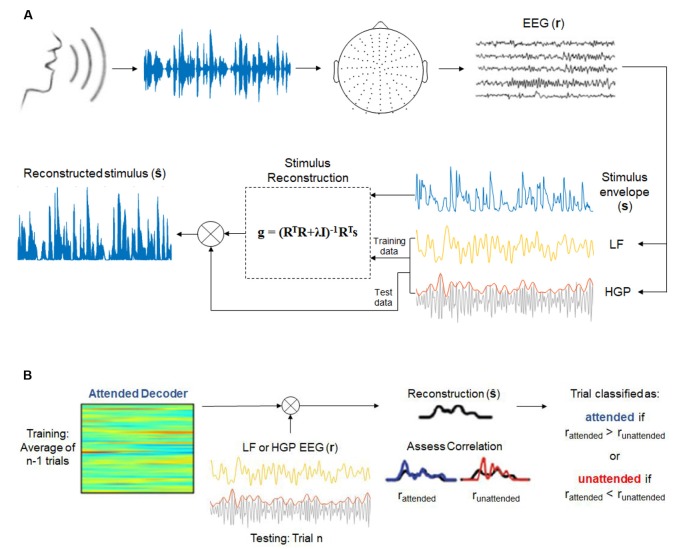
**(A)** Envelope reconstruction methods (adapted from Di Liberto et al., 2015 and Crosse et al., 2016). One-hundred and twenty-eight-channel electroencephalography (EEG) data were collected while subjects listened to a continuous, natural speech from a male speaker. Stimulus reconstruction (backward modeling) was used to decode the speech envelope from low frequency (LF, 1–15 Hz) and high gamma power (HGP, 70–150 Hz) EEG recordings. **(B)** Decoding attention methods (adapted from O’Sullivan et al., 2015 and Teoh and Lalor, 2019). Attended decoders (for LF and HGP) were made to reconstruct the attended stimuli. The correlation between the reconstructed stimulus and the attended and unattended speech envelopes were assessed.

Our analyses for both experiments were based on assessing how strongly the speech signal was represented in our different EEG bands by reconstructing an estimate of the speech envelope from the neural data (Crosse et al., 2016). Speech envelopes were extracted from the stimuli using a gammachirp auditory filterbank which mimics the filtering properties of the human cochlea (Irino and Patterson, 1997). Afterward, the envelopes were normalized between 0 and 1, and the EEG data were *z*-scored. A backward model (decoder) was employed to reconstruct the speech envelope, *s(t)*, from the neural response, *r(t, n)*, while the decoder, *g(τ, n)*, acted as a linear map between the two. The transformation can be expressed as:

$$\hat{s}(t)=\sum_{n}\sum_{\tau }r(t+\tau ,n)g(\tau ,n),$$

where $\hat{s}(t)$ is the reconstructed speech envelope. The decoder integrates the EEG (with *n* electrodes) over a range of time lags, τ, from 0 to 250 ms, the range where low-level speech features (e.g., envelope, spectrogram, and phonemes) cause notable EEG responses to occur (Di Liberto et al., 2015). The decoder (Figure 1A) was computed by the following operation:

$$g={\left(R^{T}R+\lambda I\right)}^{-1}R^{T}s,$$

where *R* is the lagged time series, *T*, of the EEG data, λ is the regularization parameter, *I* is the identity matrix, and *s* is the speech envelope.

Model performance was assessed according to the accuracy in which the speech envelope could be reconstructed using leave-one-out cross-validation. This regression allowed for an optimal regularization parameter to be chosen without overfitting to the training data. The regularization parameter that produced the highest Pearson’s correlation coefficient between the reconstructed envelope and the actual speech envelope across trials was chosen as the optimal value. Separate decoders were created for the LF and HGP groups. A model was also calculated for the combination of LF and HGP (LF+HGP) signals by concatenating the two signals (each 128-channels by delays) to form one matrix of 256-channels by delays.

To decode attention, we employed a framework introduced by O’Sullivan et al. (2015; Figure 1B). Decoder models that mapped from the EEG data to the speech envelope of the attended speaker were computed for each subject and each trial. The regularization parameter was once again determined based on leave-one-out cross-validation. We could then reconstruct the stimulus envelope of a particular trial, *n*, using the average attended decoder of *n −* 1 trials. The Pearson’s correlation coefficient, *r*, was computed between the reconstructed envelope and both the actual attended and unattended stimulus envelopes. A trial was deemed correctly classified if the reconstructed envelope was more correlated with the attended envelope than the unattended envelope (*r*_attended_ > *r*_unattended_).

### Statistical Analysis

We compared envelope reconstructions between LF and HGP EEG conditions using paired *t*-tests. One-way repeated measure analysis of variance (ANOVA) tests were used to compare stimulus reconstructions between the LF, HGP, and LF+HGP EEG conditions. The resulting statistics from the ANOVAs were Bonferroni corrected to determine significance within subjects and between groups. Lastly, two-way repeated-measures ANOVA tests were conducted to examine the effect of multiple factors on envelope reconstruction accuracy.

Testing against chance was completed using permutation tests. In the envelope reconstruction analysis, a null distribution of 10,000 Pearson’s *r* values was created by finding the correlation between randomly permuted trials of predicted audio envelopes and actual audio envelopes. The true mean correlation coefficient served as the observed value of the test statistic. In the decoding attention analysis, we performed 10,000 permutations to create the null distribution, where for each trial of each permutation we randomly selected a correlation value from either *r*_attended_ or *r*_unattended_ to be assigned to bin A, and the other to bin B. The observed value of the test statistic was the percentage of trials where *r*_attended_ > *r*_unattended_. The threshold for significance and above chance performance was *p* = 0.05 for each test. All analyses were conducted in MATLAB (The MathWorks, Inc.) and SPSS (IBM SPSS Statistics).

## Results

### Though Generally Weaker Than LF EEG, HGP EEG Consistently Tracks the Speech Envelope

**Figure 2:**
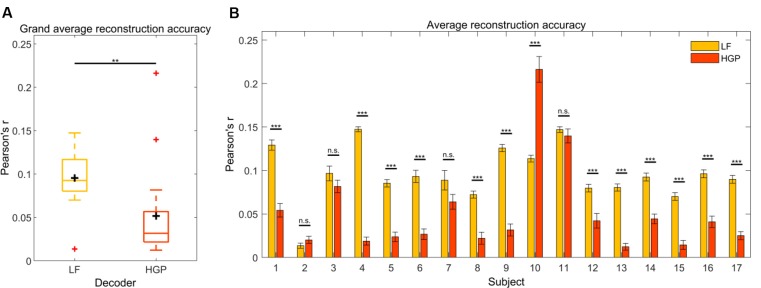
**(A)** Grand average reconstruction accuracies across trials and subjects for the LF and HGP conditions. The black crosshairs are the mean reconstruction accuracies and the red crosshairs are outliers.Significance was calculated using paired *t*-tests (**p ≤ 0.01). **(B)** Mean (±SEM) reconstruction accuracy for each subject across trials for the LF and HGP conditions. Significance was calculated using paired *t*-tests (n.s., no significance, **p ≤ 0.01, and ***p ≤ 0.001).

We first tested how well the speech envelope is reflected in LF (1–15 Hz) and HGP (power in the 70–150 Hz range) EEG. To do so, a decoder model was calculated for both conditions. Pearson’s *r* was used to quantify the relationship between the actual speech envelope and the reconstructed speech envelope. The grand average reconstruction accuracy (Pearson’s *r*) for the LF and HGP conditions were significantly larger than chance (*p* < 0.001, permutation tests, Figure 2A). Thus, on a group level, the speech envelope appeared to be encoded in the HGP EEG. Paired *t*-tests were used to assess the differences between both decoders and showed that the LF decoder reconstructed the speech envelope significantly better than the HGP decoder (*p* = 0.006).

Since recorded brain activity may vary across individuals due to anatomical differences, we wanted to examine how the LF and HGP decoders performed on a single-subject level. When tested against chance, LF and HGP were significant for all subjects (*p* < 0.05, permutation tests). As expected, the LF decoder worked best for most participants (*N* = 12, *p* ≤ 0.05, paired *t*-tests, Figure 2B). Surprisingly, there was no difference in reconstruction accuracy for subjects 2, 3, 7, and 11 (*p* ≥ 0.05). Subject 10’s LF decoder displayed a typical reconstruction accuracy, but the HGP decoder performed much better (*p* = 6.120e-08). Thus, though uncommon in EEG studies, HGP was able to track the speech envelope comparably or better than LF in some of our subjects.

To characterize factors contributing to the interindividual differences in high gamma tracking of the speech envelope, we tested whether decoding performance could be predicted from the raw power of each subject’s high gamma-band EEG. Our rationale was that perhaps subjects with stronger raw gamma power on the scalp would show better HGP tracking of the speech envelope. We calculated the average high gamma-band power of the raw EEG and averaged the power across channels and trials for each subject. Afterward, we correlated each subject’s power with their mean reconstruction accuracy using Pearson’s *r*. We found no correlation between raw high gamma-band power and HGP stimulus reconstruction accuracy (*r* = −0.043, *p* = 0.870). That said, our raw gamma power measures were acquired during stimulus presentation which was not ideal. Unfortunately, we did not have a sufficient amount of baseline (i.e., no stimulus) EEG data to determine raw gamma power in the absence of speech. Future work might consider collecting prestimulus EEG data to test if changes in raw HGP correlates with reconstruction accuracy during stimulus presentation (relative to the prestimulus baseline).

### Stimulus Reconstruction Suggests That LF and HGP EEG May Carry Complementary Information

**Figure 3:**
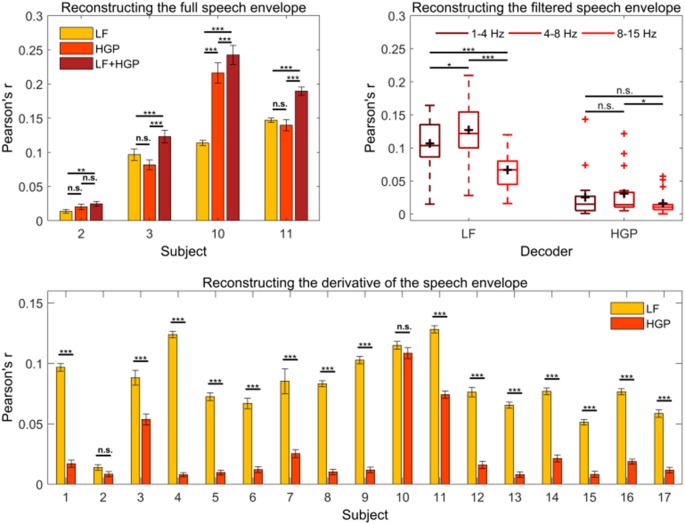
**(A)** The mean (± SEM) reconstruction accuracies for subjects 2, 3, 10, and 11 using the LF, HGP, and LF+HGP models. Significance was calculated using Bonferroni corrected one-way repeated measures analysis of variance (ANOVA) tests (n.s., no significance, *p ≤ 0.05, **p ≤ 0.01, and ***p ≤ 0.001). **(B)** Average reconstruction accuracy of filtered speech (1–4 Hz, 4–8 Hz, and 8–15 Hz bands) using the LF and HGP EEG. The black crosshairs are mean reconstruction accuracies and the red crosshairs are outliers. Significance was found using a two-way repeated-measures ANOVA test. **(C)** Syllable onset correlations of the actual speech envelope in comparison to the reconstructed speech envelope for the LF and HGP conditions. Paired *t*-tests were used to calculate significance.

Next, we tested if LF and HGP EEG carry complementary information regarding the speech envelope in subjects with comparable HGP measures. To do so, we created a combination model (LF+HGP) using 256 channels in total (128 LF EEG channels + 128 HGP EEG channels). The LF+HGP decoder had a significantly higher mean reconstruction accuracy than the LF decoder alone for subjects 2, 3, 10, and 11 (*p* < 0.05, one-way repeated measures ANOVA, Figure 3A); this suggests that HGP and LF EEG may carry complementary information. On the other hand, this could mean that LF and HGP EEG carry the same information—combining the two signals may have increased the signal-to-noise ratio of the EEG, in turn aiding the decoders’ performance.

To further examine the complementary or analogous nature of the two signals, we investigated which components of the speech envelope were being decoded from the LF and HGP EEG. The first component we examined was syllable onset. It has been suggested that syllables contain important information regarding sound segments and prosodic features of speech (Hertrich et al., 2012). Syllable onsets are approximated by computing the first derivative of the speech envelope and setting the negative values to zero (Hertrich et al., 2012). New LF and HGP decoders were trained using the derivative of the speech envelope. Single-subject results are shown in Figure 3C.

We examined the effects of speech envelope type (full envelope vs. onset envelope) and EEG band (LF EEG vs. HGP EEG) on reconstruction accuracy. We did not find a significant interaction between speech envelope type and EEG band (*p* = 0.109, two-way repeated-measures ANOVA), but we did find significant main effects of EEG band (*p* = 6.622e-06) and envelope type (*p* = 3.919e-05) on reconstruction accuracy. More specifically, utilizing LF EEG led to a higher reconstruction accuracy than HGP EEG, and the full speech envelope was reconstructed better than syllable onsets. Compared to the full envelope, decoding syllable onsets caused reconstruction accuracy to decrease by 14.88% using LF EEG and by 51.94% using HGP EEG. The decline in decoding performance suggests that HGP EEG supplies more information about the broadband speech envelope than syllable onsets, but these changes in performance between EEG band and speech envelope type were not substantial enough to result in a significant interaction effect.

Previous literature suggests that different EEG bands may reflect different functional aspects of speech processing (Cogan and Poeppel, 2011; Ding and Simon, 2014; Molinaro and Lizarazu, 2018). We reasoned that LF and HGP EEG (both in the 1–15 Hz band) may be differentially sensitive to dynamics in the speech envelope that map on to delta, theta, and alpha frequency ranges. As such, we filtered the speech envelope into 1–4 Hz, 4–8 Hz, and 8–15 Hz bands and trained new decoders on these features. The result of a two-way repeated-measures ANOVA indicated a significant interaction between EEG band and speech envelope band on reconstruction accuracy (*p* = 1.031e-04). We explored this interaction further by examining the simple main effects. The simple main effects analysis showed that the 1–4 Hz, 4–8 Hz, and 8–15 Hz speech bands were reconstructed best with LF EEG (*p* < 0.001). LF EEG reconstructed the 4–8 Hz band better than the 1–4 and 8–15 Hz bands (*p* = 0.039, *p* = 4.679e-08, Figure 3B) and the 1–4 Hz band better than the 8–15 Hz band (*p* = 2.299e-04). Alternatively, there was no difference in how well the HGP EEG reconstructed the 1–4 Hz and 4–8 Hz bands (*p* = 0.523) or the 1–4 Hz and 8–15 Hz bands (*p* = 0.470). Similar to LF EEG however, the 4–8 Hz band was reconstructed better than the 8–15 Hz band (*p* = 0.026). While this pattern of results again suggests the possibility that LF and HGP EEG are differentially sensitive to different aspects of the envelope, we are reluctant to overinterpret this, particularly in light of the generally low reconstruction scores for HGP EEG across most subjects.

### LF and HGP Responses Exhibit Different Spatiotemporal Characteristics to Speech

Our stimulus reconstruction analysis leads us to tentatively suggest that LF and HGP EEG may carry complementary information. To investigate this further, we wanted to examine if there was any evidence that our HGP and LF responses may be derived from different neural generators. To do this, we focused on subjects who showed robust HGP responses and the distribution of decoder weights across the scalp for their LF and HGP decoders. However, as decoder channel weights cannot be interpreted neurophysiologically (Haufe et al., 2014), we transformed the weights into the forward modeling space using Haufe et al.’s (2014) inversion procedure.

**Figure 4:**
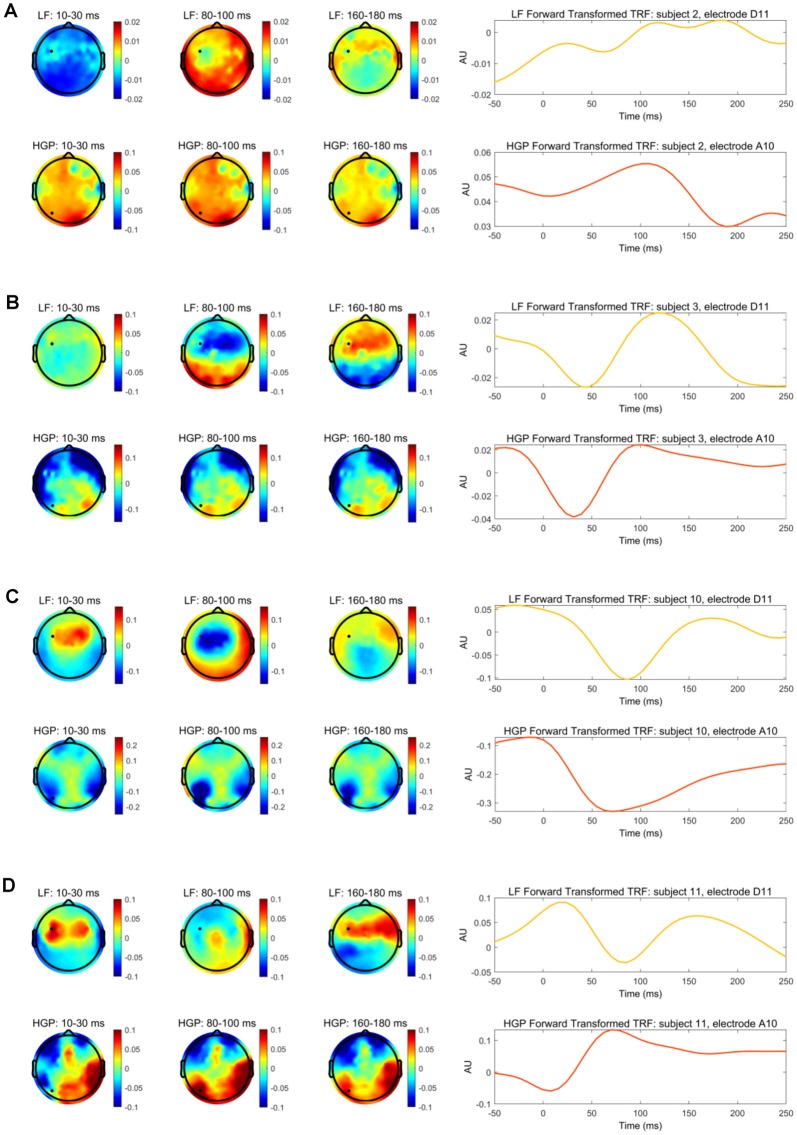
**(A)** The left panel depicts the transformed decoder weights at each electrode for subject 2. The top three topographies were calculated using LF EEG and the bottom three using HGP EEG. The black marker indicates the location for electrodes D11 and A10. The right panel shows subject 2’s temporal response function (TRF) calculated by transforming the decoder weights into the forward modeling domain. The LF TRF (yellow) is plotted for electrode D11 and the HGP TRF (red-orange) is plotted for electrode A10. **(B–D)** Same information as **(A)**, but shown for subjects 3, 10, and 11, respectively.

With this information, we compared the spatiotemporal profile of the LF and HGP EEG activity for subjects 2, 3, 10, and 11. The left panel of Figures 4A–D depicts the spatial dynamics of the forward transformed models (temporal response function or TRF) at various time lags. The LF TRF topographies appeared fairly typical (Crosse et al., 2016) as they alternated in positivity and negativity across time on the frontocentral area of the scalp (The differences in subject 2’s topographies may be due to noise and/or a DC shift in this person’s EEG data). On the other hand, their HGP activity displayed a strikingly different distribution with no prominent focus over the frontocentral scalp. These results suggest the possibility that the LF and HGP signals we see may have non-identical neural generators and further supports the idea that they may carry complementary information.

TRFs can also provide an example of how the speech envelope is transformed into neural responses over time at specific electrodes. The right panel of Figure 4 shows the forward transformed TRFs for subjects 2, 3, 10, and 11 at the electrode indicated in their topographies. The TRFs at the given electrodes are highly correlated for subject 10 (*r* = 0.872, Pearson’s correlation), moderately correlated for subjects 3 and 11 (*r* = 0.649 *r* = −0.649), and weakly correlated for subject 2 (*r* = −0.222). Once again, this weak correlation in subject 2 may be due to noisy EEG, especially since this person had very weak reconstruction accuracies. While there are differences in the time course between LF and HGP TRFs—again supporting the notion that the neural generators might differ—the general timing of the two TRFs is similar for these subjects (except subject 2) supporting the notion that the HGP TRFs, we see in these subjects are capturing real responses to our speech stimuli.

### HGP Also Improves the Decoding of Auditory Attention in Some Subjects

It has previously been shown that the LF envelope tracking response is modulated by attention (Ding and Simon, 2012) and that single-trial data from a task in which subjects attend to one of two concurrent talkers can be decoded to ascertain attentional selection (O’Sullivan et al., 2015). Given our finding that HGP contains informative temporal envelope information for a subset of subjects, we tested whether this signal is similarly modulated by attention and if it could be exploited to improve our ability to decode attention in multi-speaker situations. This was tested on a separate group of subjects (*N* = 14) from those used in the single speaker paradigm. The speakers were separated in space; the subjects attended to the speaker on the left in 50% of the trials and attended to the speaker on the right in 50% of the trials.

**Figure 5:**
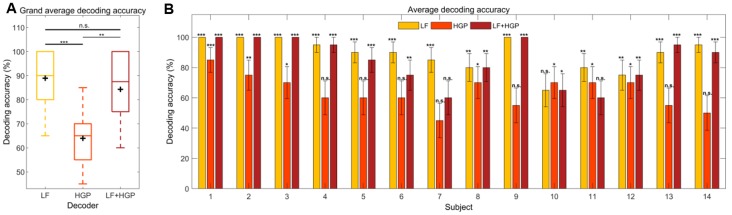
**(A)** Grand average decoding accuracies across trials and subjects for the LF, HGP, and LF+HGP decoders. The black crosshairs are the mean accuracies. Significance was calculated using a Bonferroni corrected one-way repeated measures ANOVA test (n.s., no significance, *p ≤ 0.05, **p ≤ 0.01, and ***p ≤ 0.001). **(B)** The mean (±SEM) decoding accuracies for all subjects using the LF, HGP, and LF+HGP models. Significance was calculated using permutation tests.

Here, we examine decoding accuracy which represents the percentage of trials in which the reconstructed stimuli were more correlated with the attended stream rather than the unattended stream (*r*_attended_ > *r*_unattended_) for LF, HGP, and LF+HGP EEG signals. Exploring this on a group-level showed a similar trend as our initial envelope tracking results from Figure 2A. The decoding accuracy was significantly larger than the chance for each model (*p* = 0.001, permutation test; Figure 5A). The HGP decoder was able to decode auditory attention, but it did not perform as well as the LF and LF+HGP decoders (*p* = 1.314e-04, *p* = 1.789e-03, one-way repeated measures ANOVA with Bonferroni corrections). The LF and LF+HGP decoders, however, performed similarly (*p* = 0.216).

We also examined how well we were able to decode auditory attention for individual subjects and found two whose decoding improved with HGP or LF+HGP EEG signals. Figure 5B shows the decoders that were able to track the temporal dynamics of the attended speaker significantly better than chance (*p* ≤ 0.05, permutation tests). The best attention decoding accuracy for subject 13 was achieved when using LF+HGP EEG signals (95% decoding accuracy), whereas subject 10’s best decoding accuracy was achieved using HGP EEG (70%). Interestingly, most subjects saw no improvement in decoding accuracy when LF and HGP EEG were combined, and some performed worse. The decrease in decoding performance may be due to the limited amount of EEG data available for each subject. Intuitively, given enough information, the models should learn to up-weight informative EEG channels and down-weight uninformative channels so that decoding does not worsen overall. In our case, we have included LF EEG and much noisier HGP EEG (resulting in 256 channels rather than 128) and must consider competing speech streams. The models may not have been given enough stimulus and response data to learn to down-weight the uninformative HGP EEG channels.

## Discussion

Our present research investigated the extent to which the speech envelope and attentional selection are reflected in LF phase and HGP EEG signals. Given the success of using HGP to track the dynamics of speech in ECoG studies, we wanted to examine if any useful high-frequency EEG activity remained even after being smeared by brain tissue and filtered by the skull. In this study, linear regression techniques were used to map between neural responses and the acoustic envelope of speech. Our results demonstrate that HGP EEG activity is capable of carrying information regarding the speech envelope and attention and that this information may be complementary to that of LF EEG, as shown by some of our subjects.

Previous studies using EEG techniques to study gamma band activity in the human auditory system typically examined activity around 40 Hz (Jokeit and Makeig, 1994; Krause et al., 1998; Gurtubay et al., 2001; Hald et al., 2006). A more recent study examined the high gamma range and found no significant speech envelope tracking (Viswanathan et al., 2019). This lack of success in finding/using higher frequency EEG could be because HGP is generated by highly focal sources (Jerbi et al., 2009) which in turn produce lower amplitudes on the scalp (Nunez and Srinivasan, 2006; Jerbi et al., 2009), making it more difficult to detect using EEG. This poor signal-to-noise ratio (Crone et al., 2001), along with EEG’s low spatial resolution, and high sensitivity to muscle artifacts (Llorens et al., 2011) has meant relatively few studies have focused on HGP.

In our study, we have shown that HGP, though typically weaker than LF EEG, can consistently track the temporal dynamics of speech. In some subjects, HGP EEG alone could decode the speech envelope and auditory attention best, and in others, combining LF and HGP neural signals further improved decoding. Akbari et al. (2019) also found that combining LF and HGP ECoG signals better reconstructed the speech envelope for their BCI system. Similarly, a cocktail party attention study showed that both LF phase and HGP ECoG signals can track the envelope of the attended speaker. The authors also suggested that combining the two may optimize attention encoding (Zion Golumbic et al., 2013).

The improvements in decoding/reconstruction for some subjects when LF and HGP EEG were combined—along with their notably differing topographies—suggested that LF and HGP EEG may carry complementary information. To investigate this further, we examined whether LF and HGP EEG may be sensitive to different aspects of the speech envelope. The specific features we examined were a proxy measure of syllable onsets and various frequency bands of the speech envelope. Neither analysis provided clear evidence of a dissociation, although both analyses hinted at possible differential sensitivities, with HGP being relatively less sensitive to syllable onsets and differences in 1–4 Hz vs. 4–8 Hz envelope features. Given the generally low fidelity of HGP tracking across subjects, however, we do not wish to overstate these exploratory results. Indeed, an alternative might be that LF and HGP EEG carry similar information, and their combination may improve decoding/reconstruction simply because having access to the two measures improves the signal to noise ratio of speech tracking. However, using measures of synergy, Belitski et al. showed that there is very little redundant information carried between LF and high-frequency neural signals (Belitski et al., 2010). Previous studies have also provided further support for interactions between HGP and LF phase neurophysiological signals during sensory processing (Bruns and Eckhorn, 2004; Canolty et al., 2006; Osipova et al., 2008; Voytek et al., 2010) and for the notion that speech encoding may involve the combination of the two (Nourski et al., 2009; Zion Golumbic et al., 2013). Future work either with more subjects or with both EEG and ECoG may be needed to resolve this issue.

The TRF weightings of subjects in whom we were able to detect high gamma electrophysiology displayed different spatial patterns for LF and HGP responses. HGP and LF signals are said to originate from different locations in the brain (Crone et al., 2001; Edwards et al., 2009), supporting our findings in the left panel of Figure 4. Studies also suggest that HGP is mainly localized to the superior temporal gyrus (Crone et al., 2001; Towle et al., 2008; Sinai et al., 2009) in contrast to LF activity which is more spatially distributed across temporal and some frontal and parietal regions of the brain (Crone et al., 2006; Canolty et al., 2007; Zion Golumbic et al., 2013). Although neural signals are generally spatially smeared in EEG measures, we still saw differences in the scalp patterns which may be indicative of different sources of LF and HGP activity. Of course, we need to be somewhat circumspect here, because our contrasting scalp patterns may have only arisen due to differences in the biophysics of how signals at high and low frequencies project to the scalp (Buzsáki et al., 2012). Indeed, the broadly similar timing of our LF and HGP TRFs (Figure 4) might support that notion. The abovementioned ECoG work, however, ultimately supports the idea of different generators.

We saw robust stimulus reconstruction and attention decoding using HGP EEG, but only in certain subjects. This could be attributed to hard and soft tissues filtering the source potentials, making them more difficult to measure from the scalp (Nunez and Srinivasan, 2006). Cortical folding patterns also differ between subjects which can cause certain scalp projections to vary in strength and location (Onton and Makeig, 2006). Our attempt to predict which subject would show good HGP speech tracking based on their raw HGP EEG power was unsuccessful (although, as mentioned above our measure of raw HGP EEG power could only be calculated during the presentation of the speech stimulus). A recent study found significant low gamma power EEG (in addition to delta and theta band EEG) tracking of an attended speaker across subjects but did not find the same in HGP EEG. While they showed substantial individual differences in their measures for delta, theta, and low gamma tracking, they do not report these results for HGP. This study utilized a 32-electrode cap and suggested that future studies use high-density recordings to elucidate the between-subject differences (Viswanathan et al., 2019). Here, we more fully explored individual differences in high gamma responses using high-density recordings (128-channel EEG). Future work should consider using MRI/fMRI and modeling to investigate the idea that robust HGP tracking might be related to how auditory cortex projects to the scalp in individual subjects.

In terms of the variability of HGP performance across subjects, we also noticed a few unusual patterns for specific subjects. For example, the HGP reconstruction for subject 10 was remarkably high alongside fairly typical LF tracking. This result caused us to worry initially about electrical leakage from the headphones, but there are several reasons why we are confident that this is not the case. First, we do not see high HGP in every subject, so it is not a systematic issue with our setup. Second, if the effect was coming from electrode leakage it would peak at the 0 ms delay in the TRF, which is not the case (Figure 4); the fact that the TRF peaks after 0 ms suggests it is cortical. Third, if the effect was driven by electrode leakage in just a handful of subjects (say, because of the way the cap and electrode gel were applied), it would be surprising for it to be bilateral in all of those subjects. That would suggest that the misapplication of the gel was bilateral for each subject that showed the effect, but this seems implausible. Fourth, if the effect was driven by leakage from the headphones, we might expect to see lateralization in the cocktail party data given the use of head-related transfer functions for the cocktail party attention study. We do not see this. Finally, if the effect was leakage from the electrodes, we should not see successful HGP attention decoding for any subject.

Gamma activity assessed through non-invasive means has been shown to play a role in a variety of neural processes such as working memory (Tallon-Baudry et al., 1998; Howard et al., 2003; Mainy et al., 2007; Roux and Uhlhaas, 2014), motor and sensorimotor function (Medendorp et al., 2007; Ball et al., 2008; Cheyne et al., 2008), and visual processing (Adjamian et al., 2004; Hoogenboom et al., 2006; Fründ et al., 2007). Here, we show that HGP also has value when studying speech processing and auditory selective attention—albeit in a minority of subjects. In these subjects, high gamma activity supplemented lower frequencies to increase the sensitivity to speech and attention-related processes. Therefore, it is worth investigating HGP in all subjects as this increase in sensitivity could be beneficial, for instance, for the use of future individually tuned EEG-enabled hearing devices.

## Data Availability Statement

The raw data supporting the conclusions of this article will be made available by the authors, without undue reservation, to any qualified researcher.

## Ethics Statement

The studies involving human participants were reviewed and approved by Ethics Committees of the School of Psychology at Trinity College Dublin, Health Sciences Faculty at Trinity College Dublin, and the Research Subjects Review Board at the University of Rochester. The participants provided their written informed consent to participate in this study.

## Author Contributions

ET collected the cocktail party data. SS and ET analyzed the data. SS, ET, and EL interpreted the data and wrote the article.

## Conflict of Interest

The authors declare that the research was conducted in the absence of any commercial or financial relationships that could be construed as a potential conflict of interest.

## Footnotes

^1^https://datadryad.org/stash/dataset/doi:10.5061/dryad.070jc

^2^http://www.neurobs.com

## References

[B1] Adjamian P. Holliday I. E. Barnes G. R. Hillebrand A. Hadjipapas A. Singh K. D. (2004). Induced visual illusions and γ oscillations in human primary visual cortex. Eur. J. Neurosci. 20, 587–592. 10.1111/j.1460-9568.2004.03495.x 15233769

[B2] Ahissar E. Nagarajan S. Ahissar M. Protopapas A. Mahncke H. Merzenich M. M. (2001). Speech comprehension is correlated with temporal response patterns recorded from auditory cortex. Proc. Natl. Acad. Sci. U S A 98, 13367–13372. 10.1073/pnas.201400998 11698688 PMC60877

[B3] Aiken S. J. Picton T. W. (2008). Human cortical responses to the speech envelope. Ear Hear. 29, 139–157. 10.1097/aud.0b013e31816453dc 18595182

[B4] Akbari H. Khalighinejad B. Herrero J. L. Mehta A. D. Mesgarani N. (2019). Towards reconstructing intelligible speech from the human auditory cortex. Sci. Rep. 9:874. 10.1038/s41598-018-37359-z 30696881 PMC6351601

[B5] Ball T. Demandt E. Mutschler I. Neitzel E. Mehring C. Vogt K.. (2008). Movement related activity in the high γ range of the human EEG. NeuroImage 41, 302–310. 10.1016/j.neuroimage.2008.02.032 18424182

[B6] Belitski A. Panzeri S. Magri C. Logothetis N. K. Kayser C. (2010). Sensory information in local field potentials and spikes from visual and auditory cortices: time scales and frequency bands. J. Comput. Neurosci. 29, 533–545. 10.1007/s10827-010-0230-y 20232128 PMC2978898

[B7] Broderick M. P. Anderson A. J. Di Liberto G. M. Crosse M. J. Lalor E. C. (2018). Electrophysiological correlates of semantic dissimilarity reflect the comprehension of natural, narrative speech. Curr. Biol. 28, 803.e3–809.e3. 10.1016/j.cub.2018.01.080 29478856

[B8] Bruns A. Eckhorn R. (2004). Task-related coupling from high- to low-frequency signals among visual cortical areas in human subdural recordings. Int. J. Psychophysiol. 51, 97–116. 10.1016/j.ijpsycho.2003.07.001 14693360

[B9] Buzsáki G. Anastassiou C. A. Koch C. (2012). The origin of extracellular fields and currents—EEG, ECoG, LFP and spikes. Nat. Rev. Neurosci. 13, 407–420. 10.1038/nrn3241 22595786 PMC4907333

[B10] Canolty R. T. Edwards E. Dalal S. S. Soltani M. Nagarajan S. S. Kirsch H. E.. (2006). High γ power is phase-locked to theta oscillations in human neocortex. Science 313, 1626–1628. 10.1126/science.1128115 16973878 PMC2628289

[B11] Canolty R. T. Soltani M. Dalal S. S. Edwards E. Dronkers N. F. Nagarajan S. S.. (2007). Spatiotemporal dynamics of word processing in the human brain. Front. Neurosci. 1, 185–196. 10.3389/neuro.01.1.1.014.2007 18982128 PMC2518055

[B12] Cheyne D. Bells S. Ferrari P. Gaetz W. Bostan A. C. (2008). Self-paced movements induce high-frequency γ oscillations in primary motor cortex. NeuroImage 42, 332–342. 10.1016/j.neuroimage.2008.04.178 18511304

[B13] Cogan G. B. Poeppel D. (2011). A mutual information analysis of neural coding of speech by low-frequency MEG phase information. J. Neurophysiol. 106, 554–563. 10.1152/jn.00075.2011 21562190 PMC3154802

[B14] Crone N. E. Boatman D. Gordon B. Hao L. (2001). Induced electrocorticographic γ activity during auditory perception. Clin. Neurophysiol. 112, 565–582. 10.1016/s1388-2457(00)00545-9 11275528

[B15] Crone N. E. Sinai A. Korzeniewska A. (2006). “High-frequency gamma oscillations and human brain mapping with electrocorticography,” in Progress in Brain Research. Vol. 159, eds Neuper C. Klimesch W. (Elsevier), 275–295. 10.1016/S0079-6123(06)59019-3 17071238

[B16] Crosse M. J. Di Liberto G. M. Bednar A. Lalor E. C. (2016). The multivariate temporal response function (mTRF) toolbox: a MATLAB toolbox for relating neural signals to continuous stimuli. Front. Hum. Neurosci. 10:604. 10.3389/fnhum.2016.00604 27965557 PMC5127806

[B17] Delorme A. Makeig S. (2004). EEGLAB: an open source toolbox for analysis of single-trial EEG dynamics including independent component analysis. J. Neurosci. Methods 134, 9–21. 10.1016/j.jneumeth.2003.10.009 15102499

[B18] Di Liberto G. M. Crosse M. J. Lalor E. C. (2018). Cortical measures of phoneme-level speech encoding correlate with the perceived clarity of natural speech. eNeuro 5:ENEURO.0084-18.2018. 10.1523/eneuro.0084-18.2018 29662947 PMC5900464

[B19] Di Liberto G. M. O’Sullivan J. A. Lalor E. C. (2015). Low-frequency cortical entrainment to speech reflects phoneme-level processing. Curr. Biol. 25, 2457–2465. 10.1016/j.cub.2015.08.030 26412129

[B20] Dijkstra K. V. Brunner P. Gunduz A. Coon W. Ritaccio A. L. Farquhar J.. (2015). Identifying the attended speaker using electrocorticographic (ECoG) signals. Brain Comput. Interfaces 2, 161–173. 10.1080/2326263x.2015.1063363 26949710 PMC4776341

[B21] Ding N. Simon J. Z. (2012). Emergence of neural encoding of auditory objects while listening to competing speakers. Proc. Natl. Acad. Sci. U S A 109, 11854–11859. 10.1073/pnas.1205381109 22753470 PMC3406818

[B22] Ding N. Simon J. Z. (2014). Cortical entrainment to continuous speech: functional roles and interpretations. Front. Hum. Neurosci. 8:311. 10.3389/fnhum.2014.00311 24904354 PMC4036061

[B23] Doyle A. C. (1892). “Adventure 1: a scandal in bohemia,” in The Adventures of Sherlock Holmes (London: Goerge Newnes Limited), 3–28.

[B24] Doyle A. C. (1902). The Hound of the Baskervilles. London: Goerge Newnes Limited.

[B25] Drullman R. Festen J. M. Plomp R. R. (1994). Effect of temporal envelope smearing on speech reception. J. Acoust. Soc. Am. 95, 1053–1064. 10.1121/1.408467 8132899

[B26] Edwards E. Soltani M. Kim W. Dalal S. S. Nagarajan S. S. Berger M. S.. (2009). Comparison of time-frequency responses and the event-related potential to auditory speech stimuli in human cortex. J. Neurophysiol. 102, 377–386. 10.1152/jn.90954.2008 19439673 PMC2712274

[B27] Fründ I. Schadow J. Busch N. A. Körner U. Herrmann C. S. (2007). Evoked γ oscillations in human scalp EEG are test-retest reliable. Clin. Neurophysiol. 118, 221–227. 10.1016/j.clinph.2006.09.013 17126070

[B28] Gurtubay I. G. Alegre M. Labarga A. Malanda A. Iriarte J. Artieda J. (2001). γ band activity in an auditory oddball paradigm studied with the wavelet transform. Clin. Neurophysiol. 112, 1219–1228. 10.1016/s1388-2457(01)00557-0 11516733

[B29] Hald L. A. Bastiaansen M. C. M. Hagoort P. (2006). EEG theta and γ responses to semantic violations in online sentence processing. Brain Lang. 96, 90–105. 10.1016/j.bandl.2005.06.007 16083953

[B30] Haufe S. Meinecke F. Görgen K. Dähne S. Haynes J.-D. Blankertz B.. (2014). On the interpretation of weight vectors of linear models in multivariate neuroimaging. NeuroImage 87, 96–110. 10.1016/j.neuroimage.2013.10.067 24239590

[B31] Hemingway E. (1952). The Old Man and The Sea. New York, NY: Charles Scribner’s Sons.

[B32] Hertrich I. Dietrich S. Trouvain J. Moos A. Ackermann H. (2012). Magnetic brain activity phase-locked to the envelope, the syllable onsets, and the fundamental frequency of a perceived speech signal. Psychophysiology 49, 322–334. 10.1111/j.1469-8986.2011.01314.x 22175821

[B33] Hoogenboom N. Schoffelen J.-M. Oostenveld R. Parkes L. M. Fries P. (2006). Localizing human visual γ-band activity in frequency, time and space. NeuroImage 29, 764–773. 10.1016/j.neuroimage.2005.08.043 16216533

[B34] Howard M. W. Rizzuto D. S. Caplan J. B. Madsen J. R. Lisman J. Aschenbrenner-Scheibe R.. (2003). γ oscillations correlate with working memory load in humans. Cereb. Cortex 13, 1369–1374. 10.1093/cercor/bhg084 14615302

[B35] Irino T. Patterson R. D. (1997). A time-domain, level-dependent auditory filter: the gammachirp. J. Acoust. Soc. Am. 101, 412–419. 10.1121/1.417975

[B36] Jerbi K. Ossandón T. Hamamé C. M. Senova S. Dalal S. S. Jung J.. (2009). Task-related γ-band dynamics from an intracerebral perspective: review and implications for surface EEG and MEG. Hum. Brain Mapp. 30, 1758–1771. 10.1002/hbm.20750 19343801 PMC6870589

[B37] Jokeit H. Makeig S. (1994). Different event-related patterns of γ-band power in brain waves of fast- and slow-reacting subjects. Proc. Natl. Acad. Sci. U S A 91, 6339–6343. 10.1073/pnas.91.14.6339 8022783 PMC44197

[B38] Kerlin J. R. Shahin A. J. Miller L. M. (2010). Attentional gain control of ongoing cortical speech representations in a “cocktail party”. J. Neurosci. 30, 620–628. 10.1523/JNEUROSCI.3631-09.2010 20071526 PMC2832933

[B39] Krause C. M. Korpilahti P. Pörn B. Jäntti J. Lang H. A. (1998). Automatic auditory word perception as measured by 40 Hz EEG responses. Electroencephalogr. Clin. Neurophysiol. 107, 84–87. 10.1016/s0013-4694(98)00030-3 9751279

[B40] Kubanek J. Brunner P. Gunduz A. Poeppel D. Schalk G. (2013). The tracking of speech envelope in the human cortex. PLoS One 8:e53398. 10.1371/journal.pone.0053398 23408924 PMC3542338

[B41] Lalor E. C. Foxe J. J. (2010). Neural responses to uninterrupted natural speech can be extracted with precise temporal resolution. Eur. J. Neurosci. 31, 189–193. 10.1111/j.1460-9568.2009.07055.x 20092565

[B42] Light G. A. Williams L. E. Minow F. Sprock J. Rissling A. Sharp R.. (2010). Electroencephalography (EEG) and event-related potentials (ERPs) with human participants. Curr. Protoc. Neurosci. 52, 6.25.1–6.25.24. 10.1002/0471142301.ns0625s52 20578033 PMC2909037

[B43] Llorens A. Trébuchon A. Liegeois-Chauvel C. Alario F.-X. (2011). Intra-cranial recordings of brain activity during language production. Front. Psychol. 2:375. 10.3389/fpsyg.2011.00375 22207857 PMC3246222

[B44] Luo H. Poeppel D. (2007). Phase patterns of neuronal responses reliably discriminate speech in human auditory cortex. Neuron 54, 1001–1010. 10.1016/j.neuron.2007.06.004 17582338 PMC2703451

[B45] Mainy N. Kahane P. Minotti L. Hoffmann D. Bertrand O. Lachaux J.-P. (2007). Neural correlates of consolidation in working memory. Hum. Brain Mapp. 28, 183–193. 10.1002/hbm.20264 16767775 PMC6871297

[B46] Medendorp W. P. Kramer G. F. I. Jensen O. Oostenveld R. Schoffelen J.-M. Fries P. (2007). Oscillatory activity in human parietal and occipital cortex shows hemispheric lateralization and memory effects in a delayed double-step saccade task. Cereb. Cortex 17, 2364–2374. 10.1093/cercor/bhl145 17190968

[B47] Mesgarani N. Chang E. F. (2012). Selective cortical representation of attended speaker in multi-talker speech perception. Nature 485, 233–236. 10.1038/nature11020 22522927 PMC3870007

[B48] Molinaro N. Lizarazu M. (2018). Delta(but not theta)-band cortical entrainment involves speech-specific processing. Eur. J. Neurosci. 48, 2642–2650. 10.1111/ejn.13811 29283465

[B49] Nourski K. V. Reale R. A. Oya H. Kawasaki H. Kovach C. K. Chen H.. (2009). Temporal envelope of time-compressed speech represented in the human auditory cortex. J. Neurosci. 29, 15564–15574. 10.1523/JNEUROSCI.3065-09.2009 20007480 PMC2851231

[B50] Nunez P. L. Srinivasan R. (2006). Electric Fields of the Brain: The Neurophysics of EEG. New York, NY: Oxford University Press.

[B51] O’Sullivan J. A. Power A. J. Mesgarani N. Rajaram S. Foxe J. J. Shinn-Cunningham B. G.. (2015). Attentional selection in a cocktail party environment can be decoded from single-trial EEG. Cereb. Cortex 25, 1697–1706. 10.1093/cercor/bht355 24429136 PMC4481604

[B52] Onton J. Makeig S. (2006). “Information-based modeling of event-related brain dynamics,” in Progress in Brain Research. Vol. 159, eds Neuper C. Klimesch W. (Elsevier), 99–120. 10.1016/S0079-6123(06)59007-7 17071226

[B53] Osipova D. Hermes D. Jensen O. (2008). γ power is phase-locked to posterior α activity. PLoS One 3:e3990. 10.1371/journal.pone.0003990 19098986 PMC2602598

[B54] Pasley B. N. David S. V. Mesgarani N. Flinker A. Shamma S. A. Crone N. E.. (2012). Reconstructing speech from human auditory cortex. PLoS Biol. 10:e1001251. 10.1371/journal.pbio.1001251 22303281 PMC3269422

[B55] Peelle J. E. Davis M. H. (2012). Neural oscillations carry speech rhythm through to comprehension. Front. Psychol. 3:320. 10.3389/fpsyg.2012.00320 22973251 PMC3434440

[B56] Pfurtscheller G. Cooper R. (1975). Frequency dependence of the transmission of the EEG from cortex to scalp. Electroencephalogr. Clin. Neurophysiol. 38, 93–96. 10.1016/0013-4694(75)90215-1 45909

[B57] Power A. J. Foxe J. J. Forde E.-J. Reilly R. B. Lalor E. C. (2012). At what time is the cocktail party? A late locus of selective attention to natural speech. Eur. J. Neurosci. 35, 1497–1503. 10.1111/j.1460-9568.2012.08060.x 22462504

[B58] Roux F. Uhlhaas P. J. (2014). Working memory and neural oscillations: α-γ versus theta-γ codes for distinct WM information? Trends Cogn. Sci. 18, 16–25. 10.1016/j.tics.2013.10.010 24268290

[B59] Sinai A. Crone N. E. Wied H. M. Franaszczuk P. J. Miglioretti D. Boatman-Reich D. (2009). Intracranial mapping of auditory perception: event-related responses and electrocortical stimulation. Clin. Neurophysiol. 120, 140–149. 10.1016/j.clinph.2008.10.152 19070540 PMC2819074

[B60] Synigal S. R. Teoh E. S. Lalor E. C. (2019). Including measures of high γ power can improve the decoding of natural speech from EEG. Neuroscience [Preprint]. 10.1101/785881 PMC7200998 32410969

[B61] Tallon-Baudry C. Bertrand O. Peronnet F. Pernier J. (1998). Induced γ-band activity during the delay of a visual short-term memory task in humans. J. Neurosci. 18, 4244–4254. 10.1523/JNEUROSCI.18-11-04244.1998 9592102 PMC6792803

[B62] Teoh E. S. Lalor E. C. (2019). EEG decoding of the target speaker in a cocktail party scenario: considerations regarding dynamic switching of talker location. J. Neural Eng. 16:036017. 10.1088/1741-2552/ab0cf1 30836345

[B63] Towle V. L. Yoon H. A. Castelle M. Edgar J. C. Biassou N. M. Frim D. M.. (2008). ECoG γ activity during a language task: differentiating expressive and receptive speech areas. Brain 131, 2013–2027. 10.1093/brain/awn147 18669510 PMC2724904

[B64] Viswanathan V. Bharadwaj H. M. Shinn-Cunningham B. G. (2019). Electroencephalographic signatures of the neural representation of speech during selective attention. eNeuro 6:ENEURO.0057–19.2019. 10.1523/ENEURO.0057-19.2019 31585928 PMC6873161

[B65] Voytek B. Canolty R. T. Shestyuk A. Crone N. Parvizi J. Knight R. T. (2010). Shifts in γ phase-amplitude coupling frequency from theta to α over posterior cortex during visual tasks. Front. Hum. Neurosci. 4:191. 10.3389/fnhum.2010.00191 21060716 PMC2972699

[B66] Zion Golumbic E. M. Ding N. Bickel S. Lakatos P. Schevon C. A. McKhann G. M.. (2013). Mechanisms underlying selective neuronal tracking of attended speech at a “cocktail party”. Neuron 77, 980–991. 10.1016/j.neuron.2012.12.037 23473326 PMC3891478

